# School Medical and Nursing Service in the Transvaal

**Published:** 1920-08-14

**Authors:** 


### SCHOOL MEDICAL AND NURSING SERVICE IN THE TRANSVAAL.

School medical inspection was started in the Transvaal in May 1914, but was interrupted for practically fifteen months by the war. It was restarted in 1915 on practically the present lines, and is proceeding vigorously and enthusiastically under the medical inspector, Mr. C. Louis Leipoldt, F.R.C.S., a graduate of Guy's Hospital, and his capable staff. The area comprises the whole of the Transvaal Province, bounded north by the Limpopo and south by the Vaal rivers. Its extent is greater than the combined areas of England, Scotland, and Wales. In this immense field there are some 2,000 schools and a total school population, European and native, of more than 150,000 children.

The climate of the area is excellent on the whole, but the two endemic diseases, malaria and bilharziasis, account for much of the invalidity among school-children. Rickets is practically unknown, and tuberculosis is of extreme rarity among school-children. The prevailing defects are defects of the teeth, eyes (largely owing to glare), adenoids and septic tonsils, and skin diseases. Malnutrition is not so prevalent as in the other Provinces.

Staff and organisation consists of the medical inspector, Dr. Leipoldt, and two assistant whole-time inspectors, Drs. Cleaver and Elias, a psychiatrist, Dr. Moll, three ophthalmologists, and four part-time dentists. The nursing staff consists of a nurse-superintendent, Miss Hassall, trained at the London Hospital and holding the C.M.B. certificate, and five full-time nurses (this year to be increased by four other appointments). A coloured nurse is also employed in the native schools. Nurses have far greater responsibilities than devolve upon European school nurses; within their areas they act practically independently.

Schools are visited by rota, large schools being inspected yearly and smaller ones when opportunity occurs. Necessarily much time is spent in travelling—by motor-car, railway, horse-cart, or other conveyance. Most of the schools are modern Government buildings, fully equal to those in any part of the world; a few are hired buildings employed temporarily. In malarious areas the teachers' quarters are protected, and quinine prophylaxis is carried on as in British Guiana.

### Conditions of Service.

School nurses have to be doubly qualified, possessing both maternity and general certificates, and must be bilingual, able to converse in both English and Dutch. They are graded as women teachers, with a stipend of £240, rising by increments of £10 to £300, a laundry allowance of £12 per year, and a war bonus. While on duty away from headquarters they are paid subsistence allowance at the rate of 12s. 6d. per day and travelling expenses. Nurses may average from 300 to 400 miles' travelling per month; medical inspectors average from 1,000 to 1,500 per month. School nurses must be below the age of thirty-five years, else they cannot be placed on contract; must be physically sound, and unmarried or self-supporting. They must be able to give class lessons on home nursing and hygiene and mothercraft, and are expected to give at least four such lessons per week. Owing to the wide extent of country and the difficulties of conveyance, home visiting cannot be systematically carried out, but is done in the towns.

### The Work Done.

An up-to-date clinic is being built at Johannesburg, which will serve as the headquarters of the service. Treatment elsewhere is carried out by the district surgeon or at local hospitals where such exist, or by the medical practitioners if possible. Treatment of necessitous children is entirely free. Malnourished children are fed at departmental expense at some schools.

In 1918 more than 25,000 children were examined, and more than 2,000 treated. The respective figures for 1919 are expected to be over 30,000 examined and more than 4,000 treated. The results of inspection are easily apparent in the improved cleanliness and general health in schools that have been repeatedly inspected.

A commencement has been made in educating mentally backward children by special classes, and a school for physically defective children is to be started on the High-veld. Blind and dumb children are sent down to a central institution in the Cape Province.

